# High Levels of DEAH-Box Helicases Relate to Poor Prognosis and Reduction of DHX9 Improves Radiosensitivity of Hepatocellular Carcinoma

**DOI:** 10.3389/fonc.2022.900671

**Published:** 2022-06-22

**Authors:** Xi Chen, Letao Lin, Guanyu Chen, Huzheng Yan, Zhenyu Li, Meigui Xiao, Xu He, Fujun Zhang, Yanling Zhang

**Affiliations:** ^1^Department of Minimally Invasive Interventional Therapy, Sun Yat-sen University Cancer Center, Guangzhou, China; ^2^State Key Laboratory of Oncology in South China, Sun Yat-sen University Cancer Center (SYSUCC), Guangzhou, China; ^3^Collaborative Innovation Center for Cancer Medicine, Sun Yat-Sen University Cancer Center, Guangzhou, China; ^4^Department of Interventional Radiology, Third Affiliated Hospital of Sun Yat-sen University, Guangzhou, China; ^5^Interventional Medical Center, Zhuhai People’s Hospital, Zhuhai, China; ^6^School of Laboratory Medicine and Biotechnology, Southern Medical University, Guangzhou, China

**Keywords:** liver hepatocellular carcinoma, DEAH-box RNA helicases, DHX9, radiosensitivity, DNA repair

## Abstract

**Background:**

Liver hepatocellular carcinoma (LIHC), one of the most common primary malignancies, exhibits high levels of molecular and clinical heterogeneity. Increasing evidence has confirmed the important roles of some RNA helicase families in tumor development, but the function of the DEAH-box RNA helicase family in LIHC therapeutic strategies has not yet been clarified.

**Methods:**

The LIHC dataset was downloaded from The Cancer Genome Atlas (TCGA). Consensus clustering was applied to group the patients. Least absolute shrinkage and selection operator Cox regression and univariate and multivariate Cox regression were used to develop and validate a prognostic risk model. The Tumor Immune Estimation Resource and Tumor Immune Single Cell Hub databases were used to explore the role of DEAH-box RNA helicases in LIHC immunotherapy. *In vitro* experiments were performed to investigate the role of DHX9 in LIHC radiosensitivity.

**Results:**

Twelve survival-related DEAH-box RNA helicases were identified. High helicase expression levels were associated with a poor prognosis and clinical features. A prognostic model comprising six DEAH-box RNA helicases (DHX8, DHX9, DHX34, DHX35, DHX38, and DHX57) was constructed. The risk score of this model was found to be an independent prognostic indicator, and LIHC patients with different prognosis were distinguished by the model in the training and test cohorts. DNA damage repair pathways were also enriched in patients with high-risk scores. The six DEAH-box RNA helicases in the risk model were substantially related to innate immune cell infiltration and immune inhibitors. *In vitro* experiments showed that DHX9 knockdown improved radiosensitivity by increasing DNA damage.

**Conclusion:**

The DEAH-box RNA helicase signature can be used as a reliable prognostic biomarker for LIHC. In addition, DHX9 may be a definitive indicator and therapeutic target in radiotherapy and immunotherapy for LIHC.

## Introduction

With a global increase in incidence and mortality in 2018, liver cancer was ranked as the fourth leading cause of cancer-related deaths (1). Should the current trend continue, liver hepatocellular carcinoma (LIHC), which has the highest incidence among the liver cancer types, is projected to become the third major cause of cancer-related death by 2030 (2). Over the past decade, LIHC management has significantly improved. Different types of treatment, such as resection, transplantation, local ablation, transarterial chemoembolization, and systemic therapies, are assigned according to the Barcelona Clinic Liver Cancer staging system (3). Moreover, new immunotherapy combination strategies, such as atezolizumab plus bevacizumab (4, 5), have been confirmed to improve overall survival (OS) in patients and have become a growing field in LIHC treatment. In addition, for Child-Pugh class A patients, prospective uncontrolled studies have shown that external beam radiation therapy, primarily stereotactic body radiation therapy, can achieve high rates of radiological responses with acceptable safety in tumors confined to the liver (6). Despite the many available therapeutic options, the survival rate associated with the disease remains unsatisfactory. Identifying and applying novel biomarkers can help define at-risk populations, patient prognosis, and response to therapies (7). Therefore, it is important to explore additional biomarkers to improve therapeutic strategies.

The DEAH-box RNA helicase family belongs to the superfamily 2 (SF2) helicases. They are characterized by a conserved core structure composed of two tandem RecA domains, which contain typical sequence motifs involved in RNA binding, as well as ATP binding and hydrolysis (8, 9). DEAH-box RNA helicases primarily function in mRNA metabolism, such as in mRNA splicing, mRNA nuclear export, translation, decay, transport, and storage (10). In addition, some DEAH-box helicase members, such as DHX9 and DHX33 (11), also participate in RNA sensing during innate immune activation in the presence of exogenous insults. Moreover, this family is involved in DNA repair upon damage (12). During tumor development, some members of this family, such as DHX9, DHX15, and DHX36, have been shown to be associated with tumorigenesis.

This study aimed to identify prognostic genes and risk models and to study their role in the clinicopathological characteristics, cancer cell biology, and the tumor immune microenvironment (TIM) in LIHC. To this end, we obtained a total of 15 DEAH-box RNA helicases from published literature, including DHX15, DHX33, DHX36, DHX9, DHX8, DHX16, DHX35, DHX38, DHX34, DHX29, DHX57, DHX30, DHX37, DHX40, and DQX1 (10, 13) and obtained the gene expression data and corresponding clinical data of patients with LIHC from The Cancer Genome Atlas (TCGA) database to perform further analysis with the aim of discovering improvements in radiotherapy and immunotherapy for LIHC.

## Materials and Methods

### Analysis of Differentially Expressed Genes in Tumor and Normal Tissues

#### TCGA Database

The gene expression data of normal liver and LIHC tissues was obtained from TCGA^1^. Differentially expressed prognosis-related DEAH-box RNA helicases between the tumor and normal tissue groups were analyzed using the R package “limma”, and the results were visualized as a heatmap and volcano plot (*p* < 0.05).

#### cBioPortal Database

The cBioPortal database^2^ was used to analyze the frequency of gene alterations in prognostic DEAH-box RNA helicases in LIHC.

#### Human Protein Atlas

Immunohistochemical images from different samples were downloaded from the Human Protein Atlas (HPA) database^3^ ^4^ and were used to demonstrate the translational level of DHX9 in normal and tumor tissues of LIHC.

### Single-Cell Atlas in Liver Cancer (scAtlasLC)

The scAtlasLC dataset^4^ was used to show the expression of six DEAH-box helicases in hepatocytes at the single-cell level.

### Data Collection

Gene expression data and the corresponding clinical data of 375 patients with LIHC were obtained from TCGA. The clinical information of the patients with LIHC included survival status, sex, age, tumor stage, tumor grade, stage of tumor (T), nodes (N), and metastases (M). Inclusion criteria were as follows: (1) histologically confirmed LIHC and (2) complete gene expression and clinical information. A total of 370 patients were enrolled for further analysis, all of whom had complete clinicopathological information (**Table 1**).

**Table 1:** Clinical characteristics of patients with LIHC in the study.

Variable	Number of patients	Percentage (%)
Age (years)		
≤65	232	62.7
>65	138	37.3
**Gender**		
Female	121	32.7
Male	249	67.3
**Grade**		
G1-2	232	62.7
G3-4	133	35.9
unknow	5	1.4
**Stage**		
Stage I-II	256	69.2
Stage III-IV	90	24.3
unknow	24	6.5
**T**		
T1-2	274	74.1
T3-4	93	25.1
unknow	3	0.8
**N**		
N0	252	68.1
N1	4	1.1
unknow	114	30.8
**M**		
M0	266	71.9
M1	4	1.1
unknow	100	27
**Survival status**		
Alive	240	64.9
Death	130	35.1
**Total**	370	100

T, tumor; N, node; M, metastasis.

### Identification of LIHC Subtypes Defined by Survival-Related DEAH-Box RNA Helicases

First, univariate Cox regression analysis was performed to identify prognosis-related DEAH-box RNA helicases (*P* < 0.05). Consensus clustering was then applied to systematically assess the roles and functions of DEAH-box RNA helicases. The R package “ConsensusClusterPlus” was used to assign the patients into two clusters. Kaplan–Meier (KM) curves were constructed using the R packages “survival” and “survminer” to show the OS of the two clusters. The difference in clinicopathological factors between the two clusters was analyzed through the R package “pheatmap” and shown as a heatmap.

### Establishment and Validation of a Prognostic Risk Model With DEAH-Box RNA Helicases

We used the R package “glmnet” to perform least absolute shrinkage and selection operator (LASSO) Cox regression. Based on the minimum criteria, the optimal penalty parameter lambda and corresponding coefficient criteria were determined. A prognostic model was constructed, and its risk score was calculated as follows: risk score = (gene A expression×βA) + (gene B expression×βB) …+ (gene N expression×βN), where β is the regression coefficient. A 1:1 ratio was employed to randomly group the samples into training (n = 186) and test cohorts (n = 184). Using the formula, we obtained the risk scores for all patients in the two cohorts. The median value of the risk scores in the training cohort was determined and was used to group the patients into low- and high-risk subgroups. The R packages “survival” and “survminer” were used to conduct KM survival analysis to compare the OS between the two subgroups. Time-dependent receiver operating characteristic (ROC) analyses were performed, and the area under the curve was calculated using the R package “timeROC” to assess the model. Univariate and multivariate Cox regression analyses were performed using the R package “survival” to determine the independent prognostic value of the risk score. Lastly, gene set enrichment analysis (GSEA) was used to identify the differential Kyoto Encyclopedia of Genes and Genomes (KEGG) pathways of the low- and high-risk groups. The enrichment levels and statistical significance were determined using normalized enrichment scores and nominal *p*-values, respectively.

### DEAH-Box RNA Helicases in the Context of a TIM

#### TIMER Database

In this study, the TIMER database^5^ was used to estimate the correlation between six DEAH-box RNA helicases in the risk model and the level of immune cell infiltration. We also explored the relationship between these genes and immunomodulators using the TIMER database.

#### Tumor Immune Single Cell Hub Database

We also used the Tumor Immune Single Cell Hub (TISCH)^6^ to systematically investigate the TIM heterogeneity at the single-cell transcription level.

### Cell Culture and Lentiviral Particle Transduction

The hepatocellular carcinoma cell lines Hep-3B, Huh7, and HEK293T were obtained from the American Type Culture Collection (ATCC, USA). Dulbecco’s modified Eagle’s medium was used to culture the cells with 10% fetal bovine serum and 1% penicillin/streptomycin at 37°C in a 5% CO_2_ incubator. A short hairpin RNA (shRNA) targeting the DHX9 gene was designed, and pSLenti-shRNA-DHX9#40(target sequence 5’-ACGACAATGGAAGCGGATATA-3’), pSLenti-shRNA-DHX9#41(target sequence 5’-GGGCTATATCCATCGAAATTT-3’), and pSLenti-shRNA-NC plasmids were purchased from OBiO Technology (Shanghai, China). Lentiviral supernatants were produced by transfecting HEK293T cells with lentiviral vectors (pM2.G and psPAX2) and the three plasmids using Lipofectamine 3000 transfection reagent (L3000-015; Invitrogen, USA). Hep-3B and Huh7 cells were transduced with lentiviral supernatants in six-well dishes for 24 h in the presence of polybrene and selected in the presence of 1 μg/mL puromycin for 5 d. DHX9 expression levels were examined *via* western blot analysis.

### siRNA Transfection

Hep-3B cells was passage into six-well plates. When the cell density reached 30%-50%, medium was replaced to serum-free opti-MEM medium, 750μl/well. 5uL of Lipofectamine 3000(L3000-015; Invitrogen, USA) was added to 125uL opti-MEM medium, mixed well, and placed at room temperature for 5min. 5μl siRNA (siCtrl, siDHX9-1, siDHX9-2) solution was added to 125uL opti-MEM medium and mixed well. siDHX9-1 (5′- CCCUGUCACUUGUCAGACA -3′) and siDHX9-2 (5′- GCAUGGACCUCAAGAAUGA -3′) were used in this study. SiCtrl, siDHX9-1, siDHX9-2 were purchased from Hanbio Biotechnology Co.,Ltd (Shanghai, China). After gently mixing the liquids in the above two steps, we placed it at room temperature for 15 minutes. 250 uL/well of the mixed solution was added to the cells in the 6-well plate. After being placed in the incubator for 6h-8h, the medium was replaced with a complete medium.

### Irradiation

The cell lines were X-ray irradiated at single 4 Gy and 8 Gy doses in a Rad Source model 2000PRO irradiator with a 0.3 mm copper filter and X-ray tube settings of 225 kV and 17.7 mA, dose rate of 1.78 Gy/min, and target distance of 50 cm (Rad Source Technologies, Buford, GA, USA).

### Total RNA Extraction and Quantitative Real-Time PCR

Liver cancer cell lines Hep-3B and Huh7 RNA isolation was performed using RNA extraction kit (19221ES50, Yeasen, China). RNA sample concentration was detected by a spectrophotometer (NanoDrop 3000, Thermo Fisher Scientific, Waltham, MA, USA). Take two micrograms of RNA to synthesize cDNA (5×RT Master Mix, Takara, Japan). RT-qPCR was conducted using a SYBR Green qPCR kit (Bio-rad, USA). We used glyceraldehyde-3-phosphate dehydrogenase (GAPDH) for normalization. The primer sequences were as follows:

GAPDH: F AATCCCATCACCATCTTCCAG; R AAATGAGCCCCAGCCTTC

DHX9(exon 6 A): F GGAGGAGAATGAGATTGAGTGC; R GCTTTCAGGGGAACAACATC

### Protein Extraction and Western Blot

Total protein was extracted from the cells using RIPA lysis buffer (ES0012; Yishan Biotech, Shanghai, China). Equal amounts of protein were separated on 12.5% and 8% sodium dodecyl sulfate polyacrylamide gels, which were then electrotransferred onto polyvinylidene difluoride membranes. Next, blocking was performed using 5% bovine serum albumin (BSA) for 1 h at room temperature. The membranes were incubated with primary antibodies against DHX9 (1:1000, 17721-1-AP; Proteintech, Hubei, China), DHX8 (1:10000, ab181074, abcam, UK), DHX35 (1:1000, YN4410, Immunoway, USA), DHX38 (1:1000, YN1100, Immunoway, USA), DHX57 (1:1000, #29895, Signalway Antibody, USA), GAPDH (1:10000, 60004-1-Ig; Proteintech), and γ-H2AX (1:1000, #80312; Cell Signaling Technology, Beverly, MA, USA) overnight at 4°C. After incubation with the corresponding secondary antibody at room temperature for 1 h, images were captured using the ChemiDoc™ Touching Image System (Bio-Rad, Hercules, CA, USA).

### CCK-8 Assay

Hep-3B cells were seeded into a 96-well plate with 3,000 cells per well. The 96-well plate was placed in a cell incubator. After the cells adhered to the wall, the corresponding irradiation dose(0Gy-10Gy) was given. After 5 days, the 96-well plate was taken out, the medium in the wells was aspirated, and then 100uL of the prepared CCK-8(CK04, DOJINDO, Japan) solution was added to the relevant wells (CCK-8: medium = 1:9). After that, the 96-well plate added with CCK-8 was placed in an incubator for 2 h, and then the absorbance at a wavelength of 450 nm was measured by an automatic microplate reader. Statistical data and cell proliferation curves were performed through Graphpad software.

### Colony Formation Assay

Hep-3b and Huh7 cells were seeded in a six-well plate at a density of 1000 cells/well. After irradiation of 4Gy, the cells were incubated at 37°C for 10 d, during which the medium was replaced every 3 d. The cells were fixed using cold methanol (1ml/well) and stained with crystal violet (1ml/well) for 20 min. Colonies visible to the eyes (> 50 cells) were counted.

### DNA Comet Assay

DNA damage was evaluated using a Single-cell Gel Electrophoresis Assay (Comet Assay) kit (4250050K; Trevigen, USA) according to the manufacturer’s instructions. For each sample, 80 µL of low-melting agarose gel was mixed with 20 µL of the cell suspension and spread onto comet slides, which were then immersed in alkaline electrophoresis solution. Electrophoresis was performed for 15–20 min after cell lysis and washing. The slides were neutralized for 15 min and stained with propidium iodide solution. Finally, the slides were observed under a fluorescence microscope (Nikon, Tokyo, Japan), and the DNA tail percentage was analyzed using CASP software.

### Immunofluorescence

The cells were cultured in immunofluorescent cell culture dishes prior to the experiments. After irradiation, the cells were fixed with 4% paraformaldehyde, permeabilized with 0.3% Triton X-100, and blocked with 5% BSA for 1 h. The cells were then incubated with primary antibodies against DHX9 (1:20, 17721-1-AP; Proteintech) and γ-H2AX (1:100, #80312; Cell Signaling Technology) at 4°C overnight. They were then incubated with Alexa Fluor 647-labeled (1:200, #4410; Cell Signaling Technology) and 594-labeled (1:200, #8889; Proteintech) secondary antibodies for 1 h at room temperature. After the cells were stained with 4’,6-diamidino-2-phenylindole solution, images were captured using a laser scanning confocal microscope (Olympus Optical, Tokyo, Japan).

### Statistical Analysis

GraphPad Prism 5.0 and R statistical software (version 4.1.1) were used for image processing and data analysis, respectively. KM survival analysis was performed to evaluate the prognostic differences between different clusters or cohorts based on the risk scores in LIHC. Univariate and multivariate Cox regression analyses were used to evaluate the influence of risk score on OS. Differences between the two groups were compared using a two-tailed unpaired Student’s *t*-test. Each experiment was repeated thrice. Statistical significance was set at *p < 0.05, **p < 0.01, ***p < 0.001, and ****p <0.0001.

## Results

### Identification of Prognosis-Related DEAH-Box RNA Helicases in LIHC

**Figure 1:**
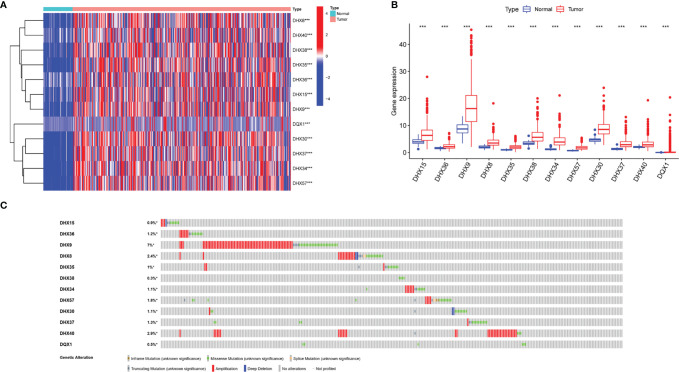
Transcriptional levels and genetic alterations of prognostic DEAH-box RNA helicases in liver hepatocellular carcinoma (LIHC). **(A)** Differential expression heatmap and **(B)** boxplot of prognostic genes in LIHC and normal tissues based on TCGA. **(C)** Gene alteration of prognosis-related helicases in LIHC based on cBioPortal data. ***p < 0.001.

The transcriptomic and clinical data of 370 patients with LIHC were downloaded from TCGA database. Based on the data, 12 prognosis-related DEAH-box RNA helicases were identified using univariate Cox regression analysis (*p* < 0.05; **Table 2**). The hazard ratio (HR) of the 12 genes was > 1, indicating a poor prognosis in patients with LIHC. Consistent with this, these 12 helicase genes were upregulated in LIHC tissues (**Figures 1A, B**). Some studies have confirmed correlations between cancer characteristics and genetic alteration signatures (14); thus, we analyzed the alterations in these prognosis-related genes using the cBioPortal database. Our findings showed that most of these genes had high mutation frequencies, with that of DHX9 being the highest. In addition, most of these alterations were amplification (**Figure 1C**).

**Table 2:** Univariable Cox regression analysis for screening prognostic genes.

gene	HR	HR.95% CI Low	HR.95% CI High	*P*-value
DHX15	1.082	1.031	1.136	0.001
DHX36	1.193	1.019	1.397	0.029
DHX9	1.037	1.017	1.058	<0.001
DHX8	1.123	1.048	1.203	<0.001
DHX35	1.312	1.100	1.564	0.003
DHX38	1.097	1.036	1.162	0.002
DHX34	1.163	1.095	1.234	<0.001
DHX57	1.453	1.217	1.735	<0.001
DHX30	1.124	1.059	1.192	<0.001
DHX37	1.223	1.123	1.332	<0.001
DHX40	1.112	1.043	1.186	0.001
DQX1	1.086	1.012	1.166	0.022

HR, hazard ratio; CI, confidence interval.

### Subtype Defined by Survival-Related DEAH-Box RNA Helicases of LIHC

**Figure 2:**
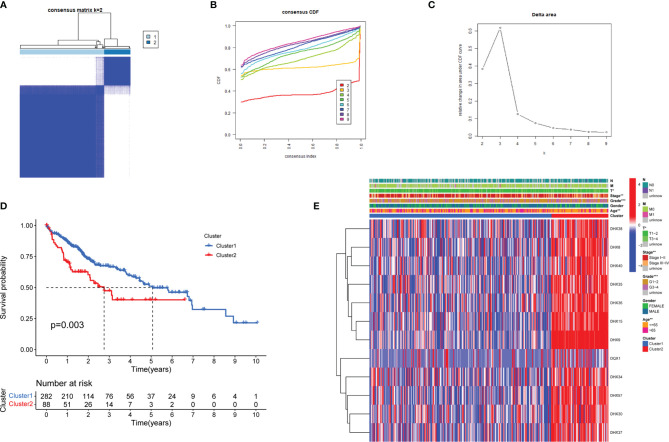
Identification of LIHC molecular subtypes based on the prognostic DEAH-box RNA helicases. **(A)** Consensus matrix heatmaps (k = 2) of prognostic genes. **(B)** Curve of cumulative distribution function (CDF) (k = 2 to 9). **(C)** The relative variation of the area under the CDF curve (k = 2 to 9). **(D)** KM curves of overall survival in the two LIHC clusters. **(E)** Heatmap of the expression of 12 survival-related genes in the different clusters and clinicopathological characteristics of the two subtypes. *p < 0.05; **p < 0.01; and ***p < 0.001.

To systematically assess the roles and functions of DEAH-box RNA helicases, the patients with LIHC were grouped using consensus clustering analysis. Based on the expression profiles of these prognostic helicases, the patients were classified into two clusters (k=2, **Figures 2A–C**). The results showed that patients in Cluster 1 tended to have longer survival than those in Cluster 2 (*p* < 0.01; **Figure 2D**). Clinical characteristics, such as age, tumor, stage, and grade, also differed between Clusters 1 and 2 (*p* < 0.01; **Figure 2E**), which suggests that these RNA helicases have a significant prognostic value in LIHC.

### Establishment and Validation of an Independent Prognostic Risk Model

**Figure 3:**
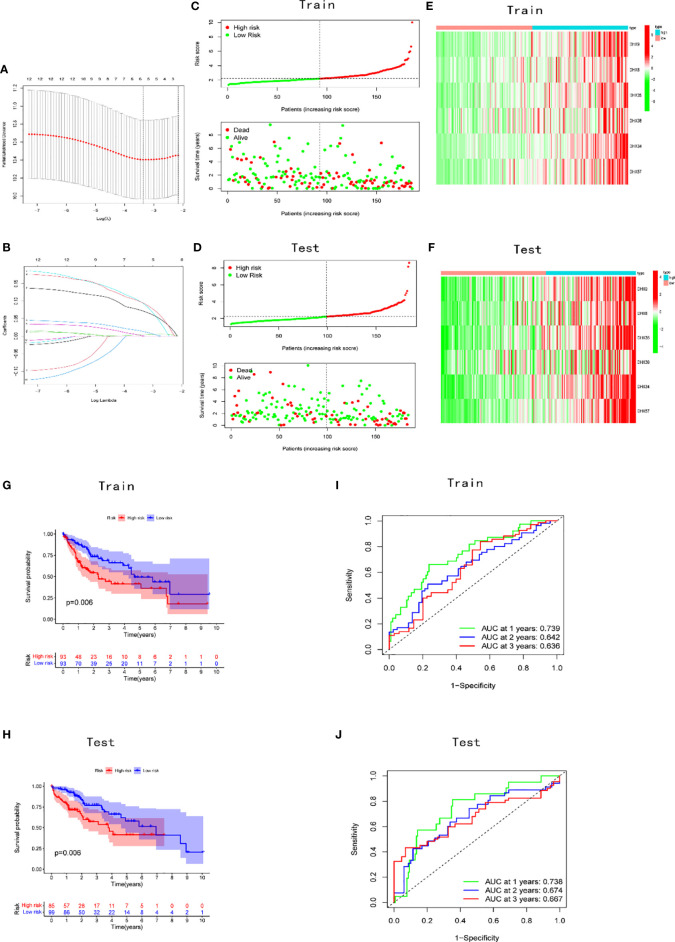
Construction and validation of the prognostic signature. **(A)** Construction of the LASSO regression model. The optimal log λ value is shown as a vertical dotted line. **(B)** The LASSO coefficient profile of DEAH-box RNA helicases, with each line representing an independent DEAH-box RNA helicase. Distribution of risk scores and prognostic status in the **(C)** training and **(D)** validation cohorts. Expression levels of the six genes in the **(E)** training and **(F)** test cohorts. KM curves in the **(G)** training and **(H)** test cohorts. Time-dependent receiver operating characteristic curve of the prognostic model in the **(I)** training and **(J)** test cohorts.

A prognostic risk model was constructed using LASSO regression for an improved prediction of prognosis of patients with LIHC. A total of 370 patients with LIHC were randomly divided into two groups, namely the training (186 patients) and test cohorts (184 patients), in a 1:1 ratio. Based on the expression values in the training cohort, 12 survival-related DEAH-box RNA helicases were used to build the prognostic model. In accordance with the minimum criteria (**Figures 3A, B**), we built a risk model comprising DHX9, DHX8, DHX34, DHX35, DHX38, and DHX57. The risk score of each patient in TCGA training and test cohorts was calculated as follows: risk score = (0.0041 × EXPDHX9) + (0.0136 ×EXPDHX8) +(0.0951 ×EXPDHX35) + (0.0009 × EXPDHX38) + (0.0722 × EXPDHX34) + (0.0955 × EXPDHX57).

**Figure 4:**
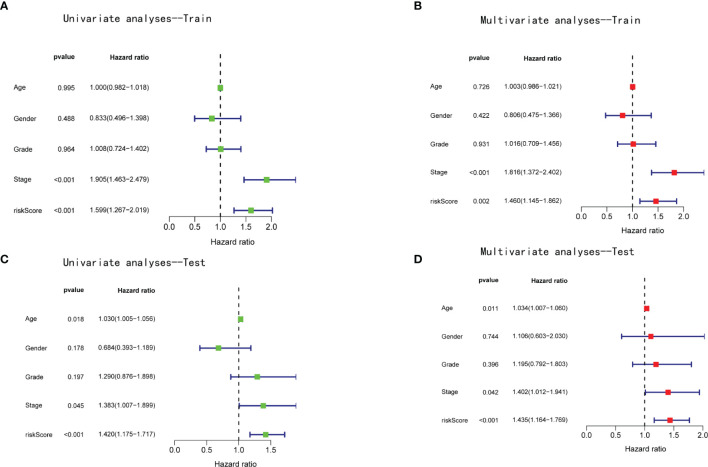
The risk score of the model was an independent prognostic factor for patients with LIHC. Univariate Cox regression analyses in the **(A)** training and **(B)** validation cohorts. Multivariate Cox regression analyses in the **(C)** training and **(D)** validation cohorts.

Patients in the training and test cohorts were divided into high-risk and low-risk groups according to the median risk score in the training cohorts. As shown in **Figures 3C, D**, fewer deaths and longer survival were observed in the low-risk group in both cohorts. We observed an upregulation of six risk genes in high-risk patients (**Figures 3E, F**). KM survival curves also indicated a worse OS in high-risk patients than in low-risk patients in both the training and test cohorts (*p* < 0.01; **Figures 3G, H**). Moreover, this six-gene risk model had a promising predictive potential for OS in both cohorts, which was demonstrated through ROC curves (**Figures 3I, J**). Next, from the results of the univariate Cox analysis in the two cohorts, we found that both risk score (training: *p* < 0.001; test: *p* < 0.001) and stage (training: *p* < 0.001; test: *p* = 0.045) were significantly associated with OS (**Figures 4A, B**). In multivariate Cox regression analyses, the risk score (training: *p* = 0.002; test: *p* < 0.001) and stage (training: *p* < 0.001; test: *p* = 0.042) were shown to be independent risk factors for patients with LIHC (**Figures 4C, D**). Furthermore, we measured the proportions of some causative agents in both the training and the test cohorts, including the history of alcohol abuse, hepatitis virus infection and nonalcoholic fatty liver disease (NAFLD). The results showed in **Tables 3**, **4**. We found that the above three factors were not statistically different between the high-risk and low-risk groups. From this we speculate that the DEAH-box helicases may be less influenced by different HCC causative agents. To explore the expression of six DEAH-box helicases in the model at the single-cell level, we searched the scAtlasLC dataset (15–18) and found that the six DEAH-box helicases were highly expressed in malignant hepatocytes (**Figure S1**). These results suggest that the 6-DEAH-box RNA helicase risk model may be useful for the evaluation of clinical prognosis.

**Table 3:** Proportion of each causative agents in training cohorts.

	level	Overall	High Risk	Low Risk	*P*-value
Alcohol (%)	(-)	122 (69.7)	62 ( 72.1)	60 ( 67.4)	0.611
	(+)	53 (30.3)	24 ( 27.9)	29 ( 32.6)	
Hepatitis_B (%)	(-)	120 (68.6)	60 ( 69.8)	60 ( 67.4)	0.863
	(+)	55 (31.4)	26 ( 30.2)	29 ( 32.6)	
Hepatitis_C (%)	(-)	141 (80.6)	72 ( 83.7)	69 ( 77.5)	0.399
	(+)	34 (19.4)	14 ( 16.3)	20 ( 22.5)	
NAFLD (%)	(-)	169 (96.6)	82 ( 95.3)	87 ( 97.8)	0.438
	(+)	6 ( 3.4)	4 ( 4.7)	2 ( 2.2)	

**Table 4:** Proportion of each causative agents in test cohorts.

	level	Overall	High Risk	Low Risk	P-value
Alcohol (%)	(-)	112 (63.6)	52 (62.7)	60 (64.5)	0.92
	(+)	64 (36.4)	31 (37.3)	33 (35.5)	
Hepatitis_B (%)	(-)	127 (72.2)	60 (72.3)	67 (72.0)	1
	(+)	49 (27.8)	23 (27.7)	26 (28.0)	
Hepatitis_C (%)	(-)	154 (87.5)	76 (91.6)	78 (83.9)	0.189
	(+)	22 (12.5)	7 (8.4)	15 (16.1)	
NAFLD (%)	(-)	162 (92.0)	77 (92.8)	85 (91.4)	0.787
	(+)	14 (8.0)	6 (7.2)	8 (8.6)	

**Figure 5:**
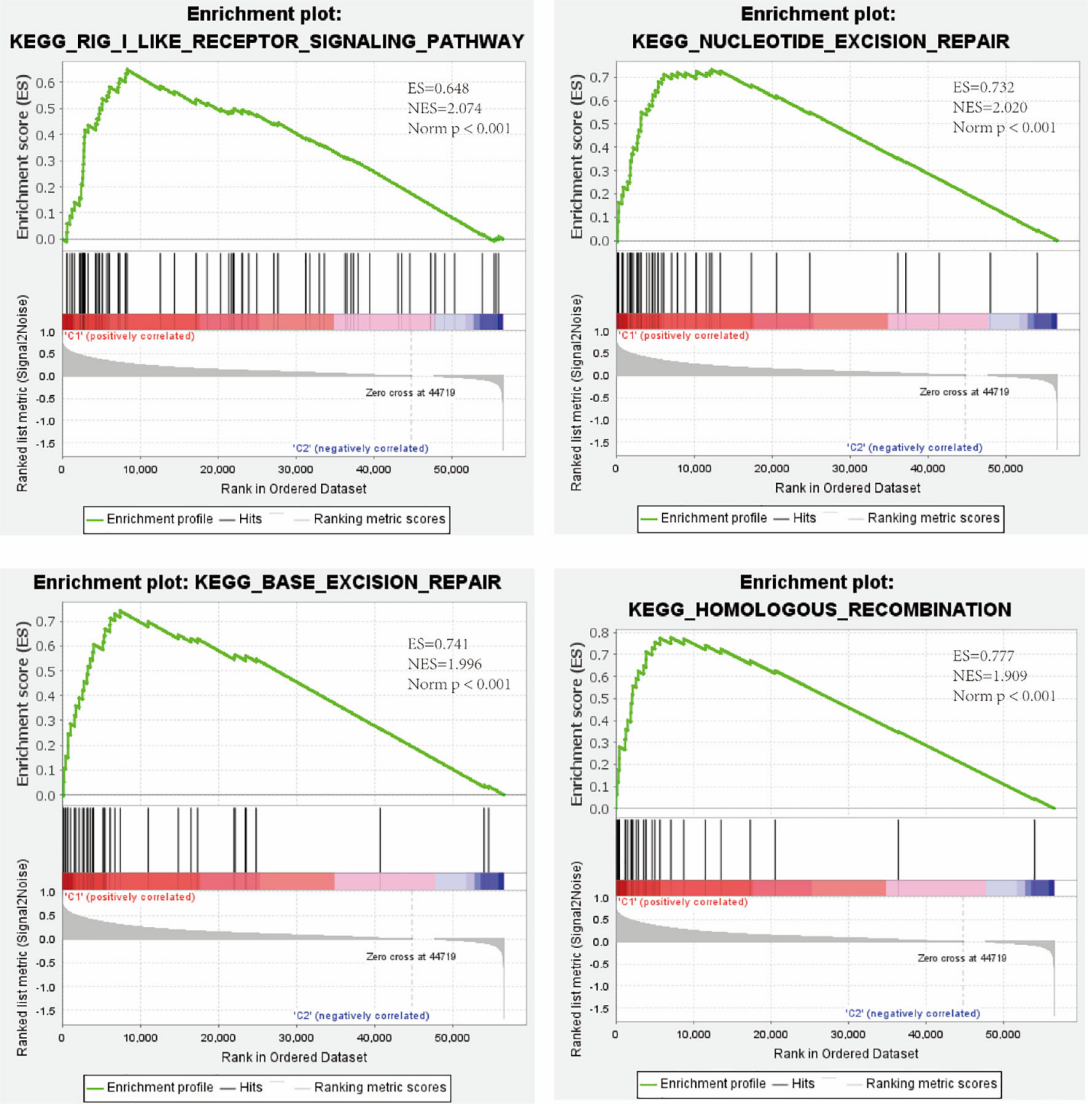
Enrichment of the RIG-I-like receptor signaling pathway and three types of DNA repair pathways in the high-risk group as revealed by GSEA. ES, enrichment score; NES, normalized enrichment score; Norm *p*, Nominal p-value.

KEGG pathway enrichment analysis of high- and low-risk patients in both cohorts was performed using the GSEA software. The results revealed that the high-risk group was positively correlated with the RIG-I-like receptor signaling pathway. Moreover, three types of DNA repair pathways, particularly nucleotide excision repair, base excision repair, and homologous recombination, were also enriched in the high-risk group (**Figure 5**).

### Correlation Between TIM and DEAH-Box RNA Helicases

KEGG pathway enrichment analysis revealed that the RIG-I-like receptor signaling pathway, which is important in the innate immune response to RNA virus infection (19), was enriched in the high-risk group. Previous studies have shown that DEAH-box helicases participate in RNA sensing in the process of innate immune activation in the presence of exogenous insults (20). As most innate immune cells play cancer-promoting roles in tumor development (21–23), we further explored the correlation between specific innate immune cells and the expression levels of six DEAH-box RNA helicases included in the risk model for LIHC. Using the TIMER database, the expression levels of the helicases were found to be significantly positively correlated with immune cells, with the correlation with myeloid-derived suppressor cell infiltration being the most evident (**Figure S2**). Furthermore, in the TISCH database, the expression levels of the six helicases were high primarily in proliferative T cells, monocytes/macrophages, and dendritic cells. We also found that DHX9 expression was the highest in immune cells (**Figure S3A**). The GSE140228_Smartseq2 dataset composed of 10 cell types was then used to analyze the distribution of the six DEAH-box RNA helicases in different immune cell types (**Figures S3B, C**). Consistent with the results shown in **Figure S2A**, the expression of DHX9 in TME-related cells was the highest, whereas that of DHX57 was the lowest (**Figure S3D**). We then explored the relationship between the six DEAH-box RNA helicases and immune inhibitors in patients with LIHC using the TIMER database. As shown in **Figures S4A–F**, the expression levels of the six DEAH-box RNA helicases were significantly positively correlated with PDCD1, CTLA4, PDCD1LG2 (PD-L1), and LAG3. These results indicate that DEAH-box RNA helicases may be closely related to innate immune cells in the TIM of LIHC.

### Role of DHX9 in the Radiosensitivity of LIHC

**Figure 6:**
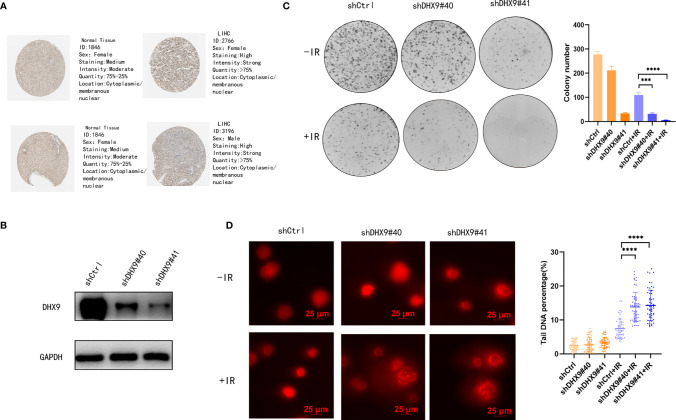
Role of DHX9 in radiosensitivity of Hep-3B cells. **(A)** Immunohistochemistry of DHX9 protein in normal and tumor tissues from the HPA database. **(B)** Western blot analysis of DHX9 expression in Hep-3B cells after stable transfection using lentivirus. **(C)** Representative images of the colony formation assay using Hep-3B cells and quantification of the colonies. **(D)** Representative images of DNA comets using Hep-3B cells (scale bar: 25 μm) and quantification of the DNA percentage of the comet tail (n>50 nuclei per sample). The data are presented as the mean ± standard deviation. Two-tailed, unpaired Student’s *t*-test was performed for statistical analysis. ***p < 0.001; ****p < 0.0001.

**Figure 7:**
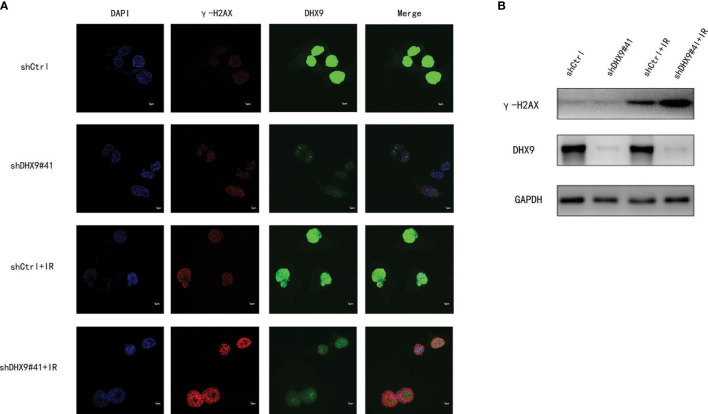
Effect of DHX9 on DNA damage repair in Hep-3B cells. **(A)** Representative fluorescence images of γ-H2AX (red) and DHX9 (green) immunostaining in Hep-3B cells (scale bar: 5 μm). Nuclei were stained with DAPI (blue). **(B)** Western blot analysis of γ-H2AX expression in Hep-3B cells. DAPI, 4’,6-diamidino-2-phenylindole.

As shown in **Figure 5**, several DNA repair pathways were also enriched in the high-risk group, and it is well known that radiotherapy (RT) has been increasingly used for the treatment of LIHC (24). Therefore, improving radiosensitivity is important. DNA damage repair (DDR) pathways, which are strongly associated with radiosensitivity (25), were enriched in the high-risk group, and DEAH-box RNA helicases were upregulated in this group; therefore, we examined the relationship between DEAH-box RNA helicases and radiosensitivity. Among the 12 survival-related RNA helicases, DHX9 had the highest expression level and amplification mutation frequency in liver tumors (**Figures 1B, C**). In addition, DHX9 has been confirmed to be involved in DDR in other cancer types (26). Therefore, we explored the effects of DHX9 on DDR and radiosensitivity in liver cancer. First, DHX9 protein expression was explored using the HPA database. Immunohistochemistry showed that DHX9 was moderately expressed in normal liver tissue and highly expressed in HCC (**Figure 6A**). DHX9 knockdown was performed and validated in Hep3B and Huh7 cell lines, and the knockdown efficacy of shRNA and siRNA was confirmed by western blot analysis (**Figures 6B**, **S5A**, **S6A**). CCK-8 assay was conducted to show the inhibitory effect of DHX9 knockdown on Hep-3B cells viability at different irradiation dose. The results showed that transfection of two DHX9 siRNAs could affect the viability of Hep-3B cells under irradiation conditions, and the effect of siDHX9-3 was greater (p<0.0001). At dose of 8gy, the difference between control group and the knockdown group was the largest (**Figure S5B**). The results of the colony formation assay indicated that DHX9 knockdown inhibited colony formation in Hep-3B and Huh7 cells, and this inhibitory effect was more significant when combined with irradiation (4 Gy) **(** **Figures 6C**, **S6B**). To determine whether DHX9 regulates DDR, we performed an alkaline comet assay, and the comet tail DNA percentage was measured to estimate the overall DNA damage. As shown in **Figures 6D** , **S6C** the DNA percentage in the comet tail significantly increased in DHX9 knockdown cells after irradiation (8 Gy), whereas DHX9 knockdown alone did not exhibit an effect as pronounced. γ-H2AX foci, which are at the center of cellular responses to DSBs, have been found to be a predictor of cell radiosensitivity (27). In our study, γ-H2AX foci detected by immunofluorescence analysis increased significantly in DHX9 knockdown Hep-3B cells after irradiation (8 Gy), suggesting that the cells were unable to efficiently repair DNA damage upon DHX9 knockdown (**Figure 7A**). The increased accumulation of γ-H2AX was further confirmed by western blot in DHX9 knockdown cells after irradiation (8 Gy) **(** **Figures 7B**, **S6D**). The results showed that the increase in radiosensitivity due to DHX9 knockdown was achieved by increasing DNA damage. Moreover, DHX9 knockdown had no effect on other four DEAH-box RNA helicases expression, namely DHX8, DHX35, DHX38 and DHX57(**Figure S7**), which indicated that DHX9 might contribute to radiosensitivity independently of other proteins in the risk model.

However, as previously reported, in Ewing Sarcomas cell lines (SK-N-MC cells), irradiation induced a new isoform of the RNA helicase DHX9, which was targeted to nonsense-mediated decay (NMD) and caused downregulation of DHX9 expression (28). In LIHC cell lines, we also verified the effect of irradiation on DHX9 expression, the results showed that irradiation had no significant effect on the mRNA(*p*>0.05)expression level and protein expression level of DHX9 also had no evident alteration, which is not the same as the regulation in sarcoma cell lines (**Figure S8**). This suggests that the effect of irradiation on DHX9 in different cancer species may not be exactly the same.

## Discussion

LIHC has high levels of molecular and clinical heterogeneity and is therefore one of the most complicated malignant cancers (29). Although there are various treatments for LIHC, the survival rate remains unsatisfactory. Thus, there is an urgent need to find more molecular biomarkers to contribute novel insights into the management of LIHC therapeutic decisions.

DEAH-box RNA helicases are a group of proteins belonging to the SF2 RNA helicases. They have a conserved structure and participate in mRNA metabolism. Moreover, they are involved in the regulation of innate immunity and DNA repair. In terms of tumor development, some DEAH-box RNA helicases have been reported in many cancers, such as lung carcinoma, colorectal carcinoma, neuroblastoma, and breast cancer (30–33). In LIHC, DHX9, DHX32, and DHX15 play important roles in promoting cancer cell motility and proliferation, inhibiting cell apoptosis, and indicating a poor prognosis (34–36). In addition, DHX9 is also involved in promoting oncogenic circular RNA CCDC66 expression and the development of chemoresistance after oxaliplatin treatment (37). However, the function of DEAH-box RNA helicase families in LIHC therapeutic strategies remains unclear. Thus, we explored the relationship between DEAH-box RNA helicase families and treatment strategies for LIHC.

First, we confirmed that the DEAH-box RNA helicase family members were mostly overexpressed in various types of cancers and then identified 12 prognostic-related DEAH-box RNA helicases. We grouped the patients according to the expression levels of these 12 RNA helicases and compared survival time, clinical features, immune checkpoints, and KEGG pathways between the two clusters. Consistent with previous studies, the results showed that Cluster 2 patients with high helicase expression levels had a poor prognosis and worse clinical features. Similar outcomes were observed in the prognostic risk model constructed through LASSO regression analysis based on six prognostic-related DEAH-box RNA helicases. High-risk patients harbored higher expression levels of RNA helicases and shorter survival times. Univariate and multivariate Cox regression analyses demonstrated that the risk model could predict the prognosis of patients with LIHC. These data suggest biological roles and the prognostic value of the DEAH-box RNA helicase family in patients with LIHC.

Studies have shown the potential of radiotherapy in LIHC patients with small lesions that are not amenable to resection or transplantation. Improving radiosensitivity not only enhances the curative effect but also decreases normal tissue damage. DDR dysregulation affects the radiosensitivity of cancer cells to radiotherapy (38). Recently, DHX9 has been found to participate in the recruitment of BRCA1 to RNA and promote DNA end resection in homologous recombination (39), as well as prevent R-loop-associated DNA damage and be overexpressed in cancer (40). DHX36 has been found to regulate p53 pre-mRNA 3’-end processing following ultraviolet-induced DNA damage (41). In our study, homologous recombination and nucleotide excision repair, which are two types of DDR pathways, were enriched in Cluster 2 with high helicase expression levels. Because DHX9 had the highest expression level and amplification mutation frequencies in liver tumors, we explored its effect on DDR and radiosensitivity in liver cancer cell lines through colony formation, alkaline comet assay, immunofluorescence, and western blotting. The results showed that the improvement in radiosensitivity after DHX9 knockdown was achieved by increasing DNA damage. We speculate that DHX9, together with other DEAH-box RNA helicase family members, has the potential to act as an indicator and therapeutic target to increase radiosensitivity during LIHC radiotherapy.

Immunotherapy has gradually become an important treatment method for many cancer types. Some DEAH-box RNA helicases participate in the regulation of innate immune reactions. Previous studies have identified that in human macrophages, DHX33 can sense RNA and subsequently activate the NLRP3 inflammasome and trigger induction of MAVS-dependent type I IFN (42). It has also been found that, depending on MAVS, DHX9 regulates the cytokine reaction to RNA virus infection through a RIG-I-independent mechanism in myeloid dendritic cells (43). Macrophages are the primary innate immune cells, and the liver contains the largest number of resident macrophages, Kupffer cells (KCs) (44). In the HCC TIM, activated KCs promote HCC *via* production of ROS and IL-6 (21, 45). Another type of innate immune cells, myeloid-derived suppressor cells, are a key element to induce immune suppression in the TIM (22). Myeloid dendritic cells can promote immunotolerance through a variety of mechanisms (46); concomitantly, neutrophils can enhance tumor cell growth, metastasis, and angiogenesis to accelerate tumor progression (23). The results of our study demonstrate that the six DEAH-box RNA helicases in the risk model had a significant positive correlation with innate immune cells (myeloid-derived suppressor cells, macrophages, myeloid dendritic cells, and neutrophils). Moreover, at the single-cell transcription level, the results showed a positive association of DEAH-box RNA helicases with innate immune cell infiltration. Thus, the poor prognosis of LIHC may be partly due to the dysregulation of DEAH-box RNA helicases, which are associated with the upregulation of these innate immune cells in the TIM. We further explored the relationship between DEAH-box RNA helicases and inhibitory receptors as upregulation of immune inhibitors, such as PD-1, PD-L1, CTLA-4, and LAG3, can lead to poor prognosis (47). This indicates that DEAH-box RNA helicases are significantly and consistently related to immune inhibitors and may be an indicator in immunotherapy.

Despite these findings, our study had some limitations. First, we used a public database with retrospective data to validate the DEAH-box RNA helicase prognostic model. Further verification with multicenter prospective clinical data is needed. Second, the correlation between DEAH-box RNA helicases and immune cells, as well as immune inhibitors in LIHC, requires further experimental validation.

## Conclusion

Our results indicate that the DEAH-box RNA helicase signature is a reliable biomarker for the prognosis of LIHC. In addition, innate immune cells may be promoters of DEAH-box RNA helicases in the malignant progression of LIHC, and DEAH-box RNA helicases may be a reliable biomarker for immunotherapy. Moreover, DHX9, a DEAH-box RNA helicase, is related to radiosensitivity and may be an indicator and therapeutic target in LIHC radiotherapy.

## Data Availability Statement

The datasets presented in this study can be found in online repositories. The names of therepository/repositories can be found in the article. Further inquiries can be directed to the corresponding authors.

## Author Contributions

XC and L-TL equally contributed and proposed the concept and design of this study. XC, M-GX, and Z-YL performed the experiments. H-ZY and XH were responsible for data collection, abstraction, and analysis. XC and LTL drafted the manuscript. Y-LZ and F-JZ critically revised the manuscript. All authors contributed to the article and approved the submitted version.

## Funding

This study was funded by the National Natural Science Foundation of China (Grant No. 81871467; 82172045).

## Conflict of Interest

The authors declare that the research was conducted in the absence of any commercial or financial relationships that could be construed as a potential conflict of interest.

## Publisher’s Note

All claims expressed in this article are solely those of the authors and do not necessarily represent those of their affiliated organizations, or those of the publisher, the editors and the reviewers. Any product that may be evaluated in this article, or claim that may be made by its manufacturer, is not guaranteed or endorsed by the publisher.

## Footnotes

1
https://portal.gdc.cancer.gov/repository.

2
https://www.cbioportal.org .

3
https://www.proteinatlas.org.

4
https://scatlaslc.ccr.cancer.gov/#/tumor-cells .
