# Supplementary material for: High Levels of DEAH-Box Helicases Relate to Poor Prognosis and Reduction of DHX9 Improves Radiosensitivity of Hepatocellular Carcinoma
Source: Front Oncol. 2022 Jun 22;12:900671. doi: 10.3389/fonc.2022.900671 (PMC9256992; doi:10.3389/fonc.2022.900671)
Supplement: Supplementary file 1 [file DataSheet_1.pdf]

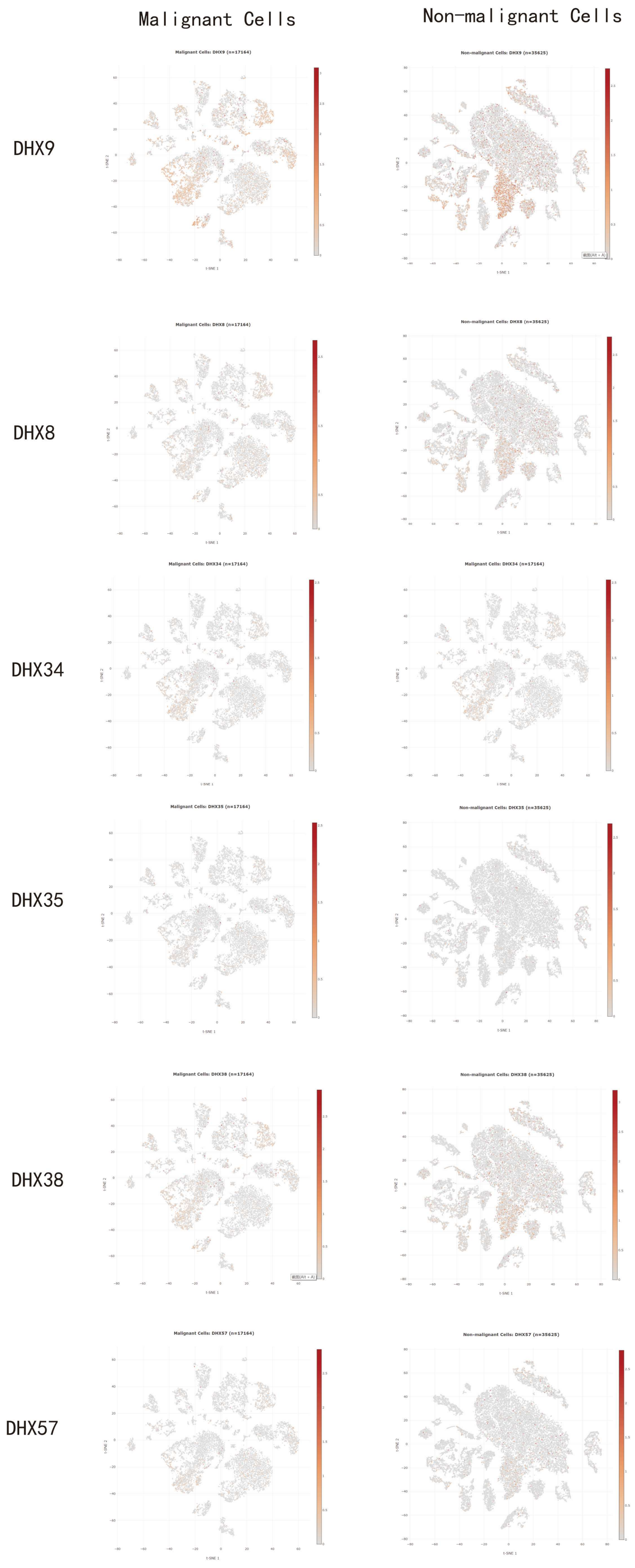

Figure S1. The expression of six DEAH-Box RNA Helicases in malignant and non-malignant hepatocytes using scAtlasLC.

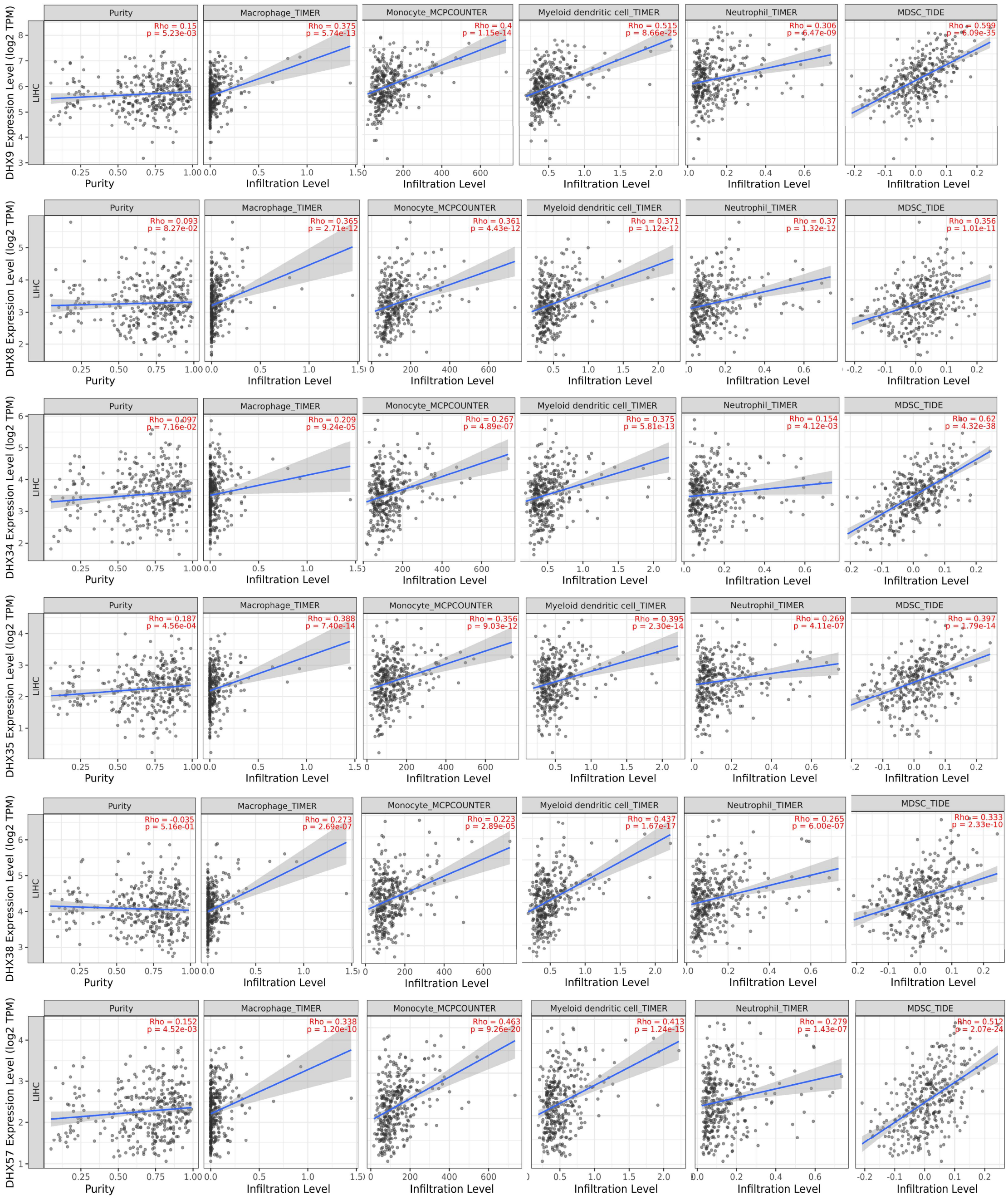

Figure S2. The relationship between the expression levels of six DEAH-Box RNA Helicases and innate immune cell infiltration using TIMER.

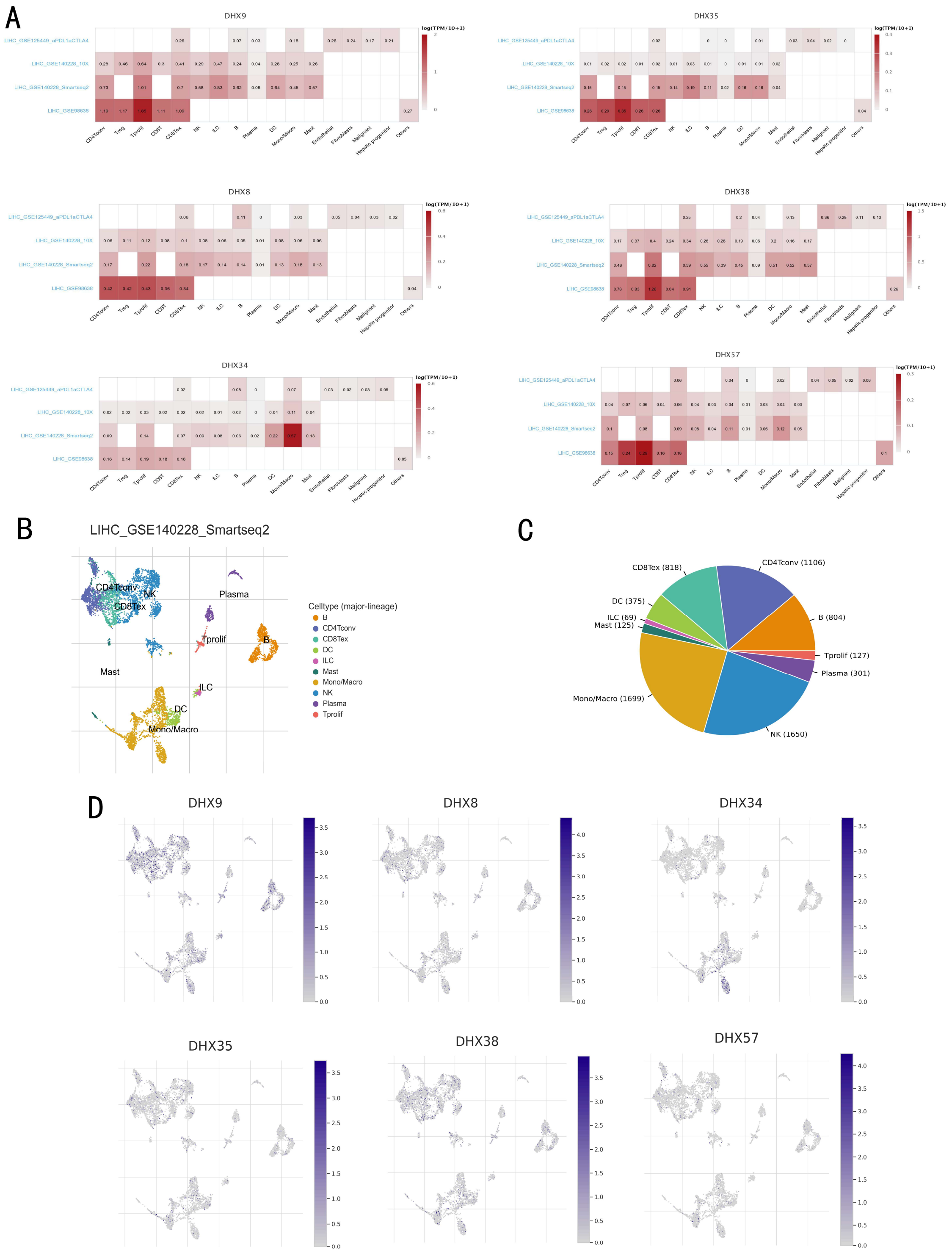

Figure S3. Correlation between the expression levels of six DEAH-Box RNA Helicases and TME-related cells type. (A) TISCH database was used to conduct correlation analysis between the expressions of DEAH-Box RNA Helicases and the TME-related cells type in LIHC. (B, C) Types and distribution of cell in the GSE140228_Smartseq2. (D) The distribution of the 6 DEAH-Box RNA Helicases in different cell types in the GSE140228_Smartseq2 dataset using single-cell resolution.

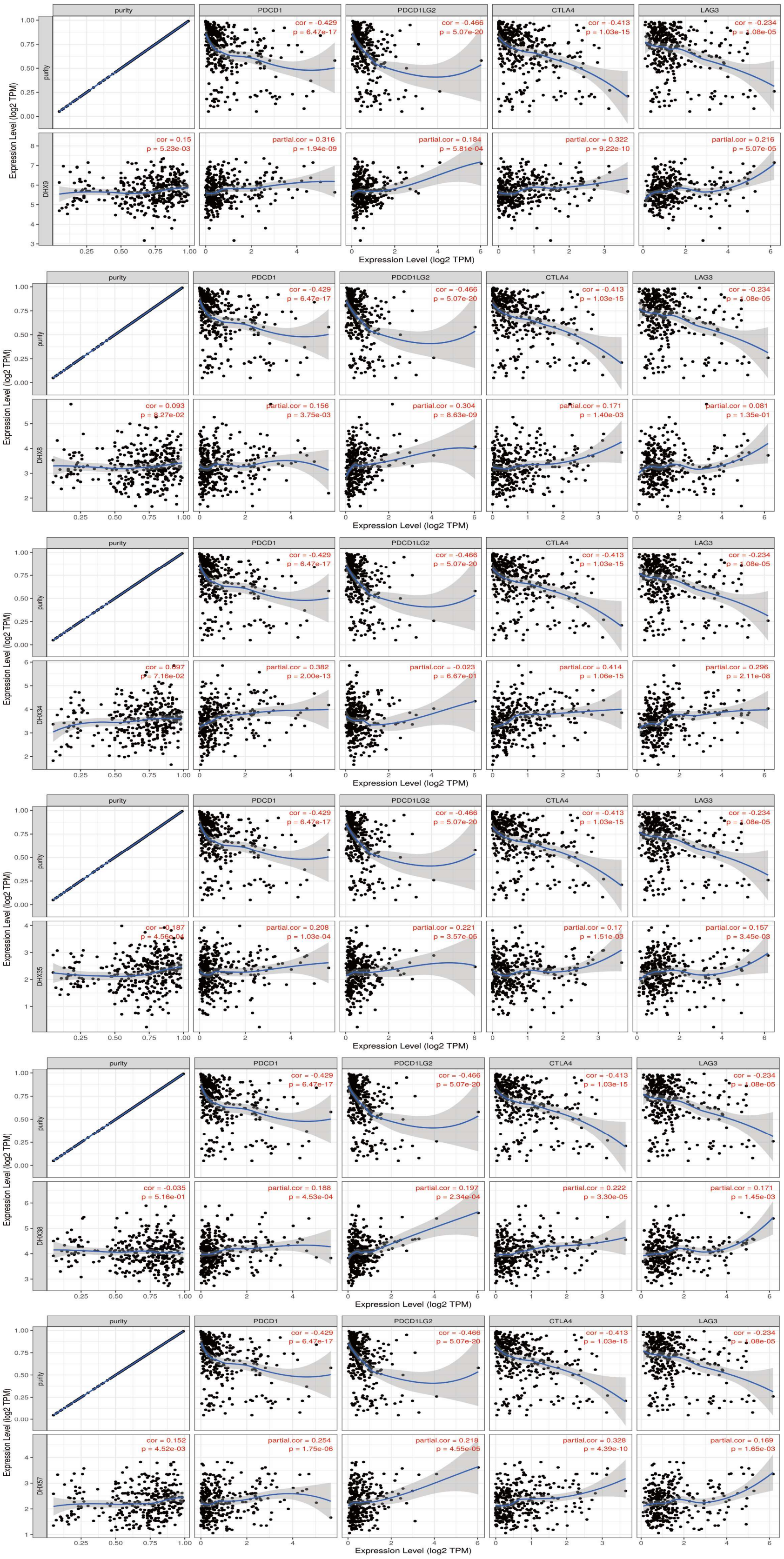

Figure S4. (A-F) The correlation between the expression levels of six DEAH-Box RNA Helicases and PDCD1, CTLA4, PDCD1LG2(PD-L1), LAG3 using TIMER.

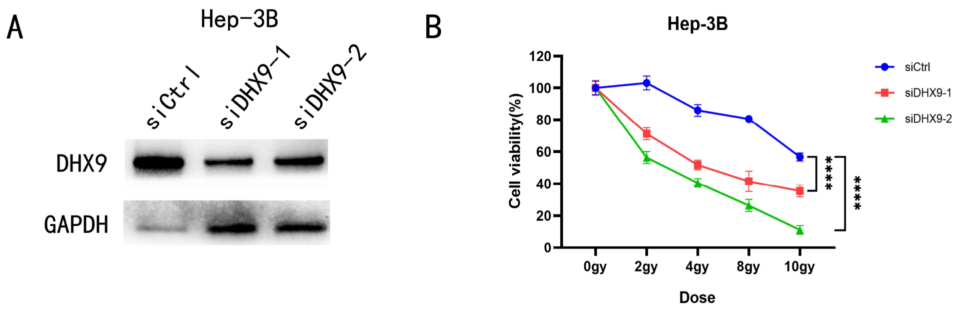

Figure S5. (A) Western blot analysis of DHX9 expression in Hep-3B cells after siRNA transfection. (B) Cell viability of Hep-3B cells 5 days later after irradiation of different dose, analyzed by CCK-8.

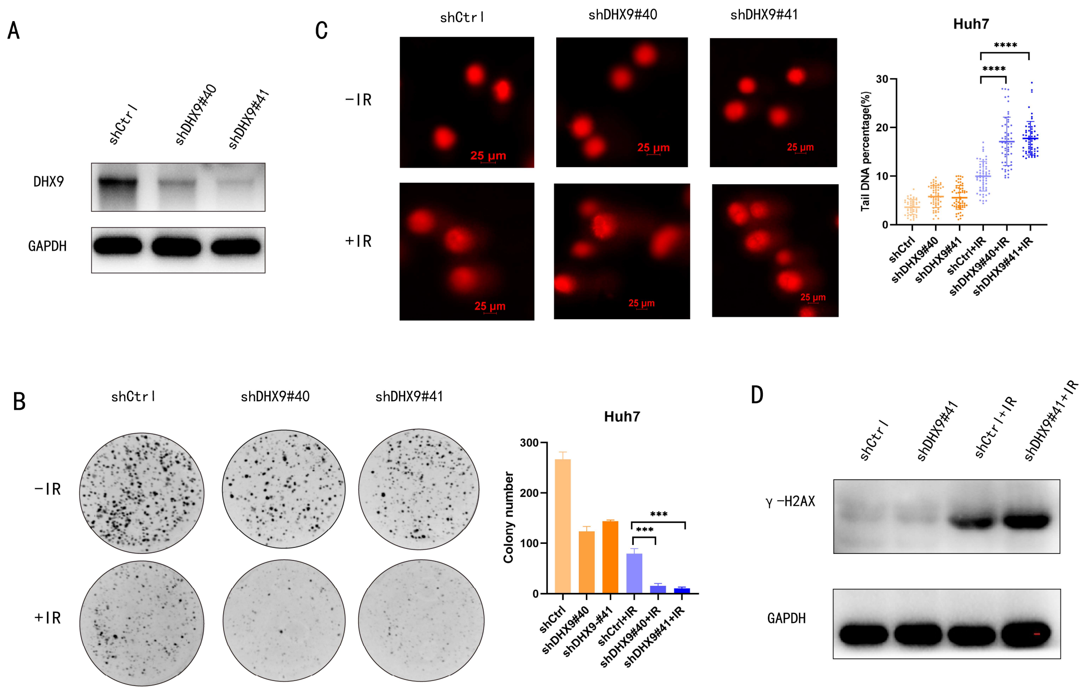

Figure S6. Role of DHX9 in radiosensitivity of Huh7 cells. (A) Western blot analysis of DHX9 expression in Huh7 cells after stable transfection using lentivirus. (B) Representative images of the colony formation assay using Huh7 cells and the quantification of the colonies. (C) Representative images of DNA comets using Huh7 cells (scale bar: 25 μm) and quantification of the DNA percentage of the comet tail (n>50 nuclei per sample). The data were presented as mean ± s.d. Two-tailed, unpaired Student's t-test was used on statistical analysis. ***$p < 0.001$; ****$p < 0.0001$. (D) Western blot analysis of expression of γ-H2AX in Huh7 cells.

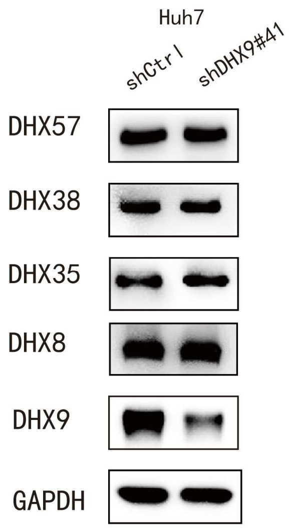

Figure S7. The effect of DHX9 on the expression of DHX57, DHX38, DHX35, DHX8.

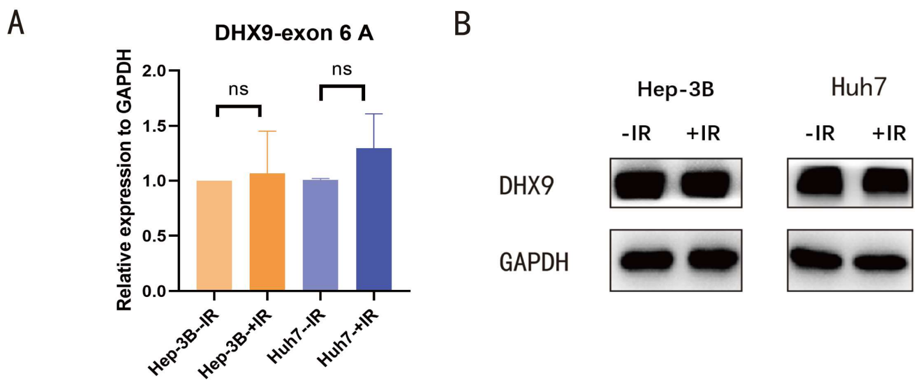

Figure S8. The effect of IR on DHX9 expression. (A) RT-qPCR and (B) WB analysis of DHX9 expression in Hep-3B and Huh7 cells with and without irradiation (8 Gy).
